# A dataset of human capital-weighted population estimates for 185 countries from 1970 to 2100

**DOI:** 10.1038/s41597-024-03466-y

**Published:** 2024-06-12

**Authors:** Guillaume Marois, Jesus Crespo Cuaresma, Jakob Zellmann, Claudia Reiter

**Affiliations:** 1 Asian Demographic Research Institute, School of Sociology and Political Sciences, Shanghai University, Shanghai, China; 2 International Institute for Applied Systems Analysis, Wittgenstein Centre for Demography and Global Human Capital (IIASA, OeAW, Wittgenstein Centre for Demography and Global Human Capital (IIASA, OeAW, University of Vienna), Laxenburg, Austria; 3 Department of Economics, Vienna University of Economics and Business (WU), Vienna, Austria; 4 Education and Employment research group, Institute for Advanced Studies (IHS), Vienna, Austria

**Keywords:** Economics, Sociology, Developing world

## Abstract

We provide a novel dataset of human capital-weighted population size (HCWP) for 185 countries from 1970 to 2100. HCWP summarizes a population’s productive capacity and human capital heterogeneity in a single metric, enabling comparisons across countries and over time. The weights are derived from Mincerian earnings functions applied to multi-country census data on educational attainment. The model used to compute the returns to schooling accounts for the diminishing positive relative relationship between education and wages as the overall education of populations rises. The population weights are adjusted by a skills assessment factor representing differences in education quality across countries and years. HCWP is calculated by applying these adjusted human capital weights to population estimates and projections disaggregated by age, sex and education, spanning the period 1970–2020 and 2020–2100 for five Shared Socioeconomic Pathway scenarios. Validation analyses demonstrate the utility of the new HCWP data in explaining national income trends. As a more comprehensive population measure than basic size and age-sex indicators, HCWP enhances the power of statistical models aimed at the assessment of socioeconomic change impacts and forecasting.

## Background & Summary

This dataset provides human capital weighted population (HCWP) estimates for 185 countries at 5-year intervals, spanning 1970–2020 historically and projected for 2020–2100 under five Shared Socioeconomic Pathways scenarios (SSP1-SSP5)^1^. The HCWP adjusts the working-age population (20–64 years) by weights based on educational attainment and education system quality. It summarizes heterogeneity in human capital in a single indicator. Potential uses include assessing past and future economic impacts of demographic and societal changes, comparing productive capacity across countries and historical periods, and predicting income growth making use of econometric models.

Traditional demographic analyses focus on population size and age-sex structure. Age structure, in particular, is an important economic growth predictor. In the decades after a fertility fall, a demographic dividend can emerge, characterized by a high working-age to dependent population ratio. This period tends to see rapid industrialization, education expansion, and economic growth^2–4^. After some decades under this regime, the elderly share of the population begins rising, bringing population aging and its challenges. The standard narrative is that a higher elderly percentage has negative economic consequences: the retired elderly population produce less but require pensions and healthcare, while the working-age share that provides support and tax revenue shrinks.

Focusing exclusively on changes in the age structure as a predictor of economic growth trends does not provide a complete picture. The ability of the ‘supporting population’ (the working-age) to actually support the ‘dependent population’ depends to a large extent on its education, labour force status and productivity, all of which are also undergoing drastic changes. Econometric models estimated on historical data have shown that the inclusion of information on education and age structure, as well as their interplay, contribute significantly to the explanatory power of statistical specifications aimed at reproducing differences in past income and income per capita trends across countries of the world. In particular, the empirical results indicate that including variables which account for age-structured educational attainment levels in economic growth models are better able to account for innovation and technology adoption phenomena^5–8^.

The Wittgenstein Centre (WIC) Population Projections provide standardized estimates and projections of population by educational attainment for all countries, spanning 1970–2100. Since working-age individuals have varying productivity, these projections use education as a proxy for human capital to enable more refined analysis of demographic change impacts^8–10^. Education correlates not just with fertility and mortality, but also labor force participation and productivity^11–16^. For example, WIC projections show India will have a larger working-age population than China in coming decades, but with much lower educational attainment and therefore less productive workers^17^. While only 13% of the working age population in China had post-secondary attainment in 2015, versus 34% in the US, this gap is projected to narrow over time as older, less-educated Chinese cohorts are replaced by more educated ones.

The role played by education as a catalyst of socioeconomic change is not only a matter of quantity but also modulated by education quality. There is ample evidence of differences in the quality of education systems among countries in the world^18^. A high school diploma does not, on average, provide the same human capital, and its effect on productivity may depend on whether it was obtained in a rich country that spends a lot on education or in a low-income country where schools are underfunded^19^.

The human capital-weighted population (HCWP) estimates presented here are calculated by applying weights to the working-age population estimates by educational attainment, which are adjusted by a factor accounting for education system quality. Following an approach similar to other studies^20,21^, education-specific weights are calculated utilizing a Mincerian earnings function^22^ on pooled data from all IPUMS-I censuses containing education and income information. The education parameters are interacted with the countries’ average educational attainment to account for the dependence of returns to education on the number of workers sharing that education level. Country and time specific adjustment factors for education quality are derived from skills assessment surveys. These final adjusted weights are then applied to population estimates and projections from the Wittgenstein Centre Data Explorer, spanning 1970–2015 historically and projected to 2100 under five Shared Socioeconomic Pathways scenarios (SSP1-SSP5).

We validate our estimates making use of an out-of-sample prediction exercise for GDP per capita growth applied to a panel dataset covering all countries of the world for which data are available. In this exercise, we estimate alternative regression specifications for the growth rate of GDP per capita based on Crespo Cuaresma^5^ and compute several prediction error statistics for the period 2000–2015 (in 5-year steps). In particular, we entertain three different econometric models: a benchmark specification where labor input is measured based on population figures, a second one which includes population by educational attainment level and a third model based on HCWP. Our results indicate that the predictive ability of the model based on HCWP is similar to that of the model based on educational attainment when measured using the global sample, and that these two specifications present a better forecasting performance than the benchmark model.

## Methods

### Estimates of education-specific weights

#### Data sources and selection of the sample

In order to derive generalizable weights linked to educational levels, we compiled all available censuses from the IPUMS-International database that included both total personal income as well as education variables. We opted to utilize total income rather than wage income alone due to the lack of wage data across many of the surveys. Given that for the majority of individuals, wages typically constitute the primary component of overall income, and the two measures are therefore highly correlated, we make the assumption that total income can serve as a reasonable proxy for wages in our analysis.

Since Mincerian wage regression models assume a log-linear relationship between wages and years of schooling, those persons not active in the formal labor force as well as those with reported incomes at or below zero were assigned an income value equal to one for modeling purposes. Employed individuals with missing income data (4.3%) were omitted from the sample altogether. The IPUMS-International census data leveraged offers the benefit of standardized definitions and categorical binning of key variables. However, the income figures are expressed in national currency units corresponding to the specific period of each survey. As such, we additionally normalized respondents’ incomes by dividing through by the national average income level in order to render the values more readily comparable across countries and time.

The education variable is provided in two formats - number of years of education or highest educational attainment level, with varying category breadth. We opted to use years of education in our models since broad attainment categories can obscure substantial within-group heterogeneity. For example, in countries with mandatory education, the lowest group comprises those lacking a high school diploma, while in developing countries surveys more typically differentiate individuals with no schooling from those with some primary or lower secondary schooling.

For surveys only reporting educational attainment, levels were converted to estimated years of schooling as follows:

- Less than primary completed → 1 year;
- Primary completed → 6 years;
- Lower secondary complete → 9 years;
- Secondary completed → 12 years;
- University completed → 16 years.

Surveys were excluded if attainment categories were insufficiently detailed for comparison to other data sources, such as a single category encompassing both high school and university completion.

We excluded individuals aged under 20 and over 64 from the sample. With the pooled censuses still comprising several million cases even after applying age criteria, we randomly selected a large enough sample from each to generate stable and robust estimates while enabling feasible computational runtimes for the regression models. The included surveys and their corresponding sample sizes are listed in Table 1.

**Table 1:** Selected censuses and their sample size.

Country	Year	Total
Canada	1971	14,107
Canada	1981	36,089
Canada	1991	60,552
Canada	2001	67,358
Canada	2011	78,992
Colombia	1973	63,321
Dominican Republic	1981	18,836
Dominican Republic	2002	39,450
Mauritius	2000	42,657
Mexico	1995	48,188
Panama	1980	21,516
Panama	1990	23,340
Panama	2010	23,842
Puerto Rico	1990	47,700
Puerto Rico	2000	52,336
Puerto Rico	2005	25,586
Puerto Rico	2010	26,416
South Africa	1996	20,606
South Africa	2001	23,427
South Africa	2007	27,417
South Africa	2011	30,203
Trinidad and Tobago	1970	28,488
Trinidad and Tobago	2000	69,792
United States	1960	118,991
United States	1970	136,357
United States	1980	64,923
United States	1990	88,255
United States	2000	99,859
United States	2005	102,436
United States	2010	111,243
United States	2015	116,360
Total		1,728,643

#### Models

We estimated the education-specific weights from Mincerian earnings function^22^, which is widely used in the literature to measure the return on investment in education^23^. We start by building a simple log-linear model with gamma distribution, predicting the natural log of normalized income (NOR_INC) from years of education (NBEDU), controlling for experience and sex, but ignoring sample design,

1 $$ln\left(NOR\_INC\right)={\beta }_{0}+{\beta }_{1}\,NBEDU+{\beta }_{2}\,EXPERIENCE+{\beta }_{3}\,EXPERIENC{E}^{2}+{\beta }_{4}\,SEX+\varepsilon $$

${\beta }_{1}$ thus provides the semi-elasticity of income to years of education. The variable EXPERIENCE is derived by subtracting from the age the number of years of education and the age of entry at school (set by default to 6 for everyone). Since we aim at estimating generalizable weights, a sample of pooled censuses from different years and different countries is used. Therefore, to account for this sample design, we built a multilevel random-effect model (model 2) which allows the intercept ${\beta }_{0}$ to vary across censuses (j).

2 $$ln\left(NOR\_INC\right)={\beta }_{0j}+{\beta }_{1}\,NBEDU+{\beta }_{2}\,EXPERIENCE+{\beta }_{3}\,EXPERIENC{E}^{2}+{\beta }_{4}\,SEX+{\varepsilon }_{j}$$

Finally, we build a final third model to account for the possible variation in the impact of years of education across countries based on the average level of education of their population^23^. This model adds parameters β_5_ and ${\beta }_{6}$ for the interaction between the number of years of education at the individual level and a country-level variable that refers to the average years of schooling for the population aged 25 to 54 (NBEDU_MEAN) in each census year.

3 $$ln(NOR{\rm{\_}}INC)={\beta }_{0j}+{\beta }_{1}\,NBEDU+{\beta }_{2}\,\ast \,EXPERIENCE+{\beta }_{3}\,EXPERIENC{E}^{2}+{\beta }_{4}\,SEX\,\,+\,{\beta }_{5}\,NBEDU{\rm{\_}}MEAN+{\beta }_{6}\,NBEDU\times NBEDU{\rm{\_}}MEAN+{\varepsilon }_{j}$$

#### Parameters

Table 2 shows the parameter estimates for the three models employed. Despite income normalization, the covariance estimates in the model given by Eq. (2) indicate statistically significant intercept variability across censuses, evidence supporting the mixed model. The introduction of an interaction term between an individual’s years of schooling and the country’s average years of schooling in the model given by Eq. (3) reveals that the positive relationship between education and wages diminishes in size as a population’s overall education level rises. Specifically, the negative parameter predicts smaller wage gains for marginal increases in an individual’s schooling within societies where higher education is more widespread. This is in line with economic principles, as the marginal value of educational attainment decreases when advanced skills become common, and thus the advantages conferred by an extra year of schooling are attenuated when competing against a highly educated population. Thus, the model given by Eq. (3) provides evidence that the labor market return of education depends on both relative and absolute skills and declines in contexts where human capital is abundant. Across all model specifications, the estimated parameters for NBEDU (years of schooling) remain remarkably consistent and comparable, underscoring the reliability of these coefficients.

**Table 2:** Parameters of models (standard error in parentheses).

	Model (1)	Model (2)	Model (3)
Random effect (group = census)		*Covariance*	*Covariance*
Intercept		0.208 (0.054)	0.202 (0.054)
	*Estimate*	*Estimate*	*Estimate*
Intercept	−3.257 (0.008)	−3.410 (0.082)	−3.350 (0.337)
NBEDU	0.135 (0.001)	0.146 (0.000)	0.213 (0.002)
NBEDU_MEAN			−0.011 (0.033)
NBEDU*NBEDU_MEAN			−0.006 (0.000)
EXPERIENCE	0.076 (0.000)	0.077 (0.000)	0.077 (0.000)
EXPERIENCE^2^	−0.001 (0.000)	−0.001 (0.000)	−0.001 (0.000)
SEX = Male	0.798 (0.003)	0.794 (0.002)	0.794 (0.002)
N	1377624	1377624	1377624
Clusters		31	31
ICC		<0.001	<0.001

Using parameters from the model in Eq. (3), we calculate relative weights (W) for each educational level (e), which vary depending on the population’s average years of schooling (NBEDU_MEAN) of the country (c) in a given year (t). We postulate that having less than primary completed (e1) corresponds to 1 year of schooling (NBEDU = 1), primary completed (e2) to 6 years, lower secondary (e3) to 9 years, upper secondary (e4) to 12 years and postsecondary (e5) to 16 years. We normalize the weights making use of the value for the world average number of years of schooling in 2015 (9.3). The calculation of W is thus given by

4 $${W}_{e,c,t}=\frac{{\rm{e}}{\rm{x}}{\rm{p}}({\beta }_{1}\ast NBED{U}_{e,c,t}+{\beta }_{5}\ast NBEDU{\rm{\_}}MEA{N}_{c,t}+{\beta }_{6}\ast NBEDU\ast NBEDU{\rm{\_}}MEA{N}_{c,t})}{{\rm{e}}{\rm{x}}{\rm{p}}({\beta }_{1}\ast 9.3+{\beta }_{5}\ast NBEDU{\rm{\_}}MEA{N}_{c,t}+{\beta }_{6}\ast 9.3\ast NBEDU{\rm{\_}}MEA{N}_{c,t})}$$

**Fig. 1:**
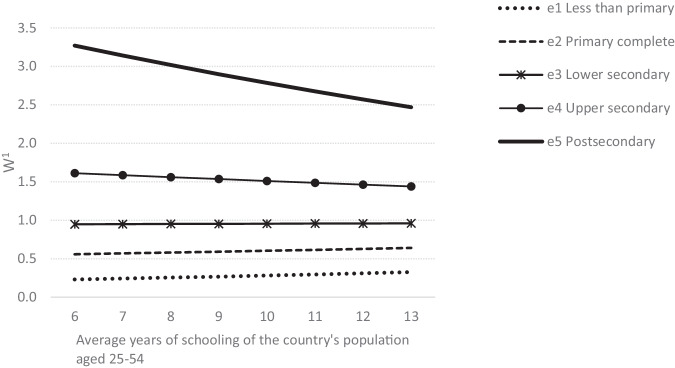
Weights (W) by educational levels and average years of schooling of the 25–54. *1* = *world average level of education* = *9.3*.

Figure 1 below presents weights by educational attainment and average years of schooling. In a country such as the US in 2020, where the average number of years of education for the population aged 25–54 was 13.2 years, a person with postsecondary education would thus be weighted 2.45 times more than a worker with the world average number of years of education and, assuming that income is a good proxy for productivity^24^, would thus be 2.45 times more productive.

### Factorizing for the quality of education

Lutz *et al*.^18^ introduced the Skills in Literacy Adjusted Mean Years of Schooling (SLAMYS) to assess the quality of education and human capital. This indicator multiplies the mean years of schooling (MYS) by a factor that takes into account the quality of education (skill adjusted factor or SAF). SAFs are empirically derived from the scores of adult literacy assessments (for individuals aged 20–64) from surveys such as the International Adult Literacy Survey (IALS), the Program for the International Assessment of Adult Competencies (PIAAC), the Skills toward Employment and Productivity Survey (STEP), and the Demographic and Health Survey (DHS). They are calculated cross-sectionally for 185 countries for the period 1970–2015 and normalised to the score for the population-weighted average of OECD countries in 2015 (taken as unity).

**Fig. 2:**
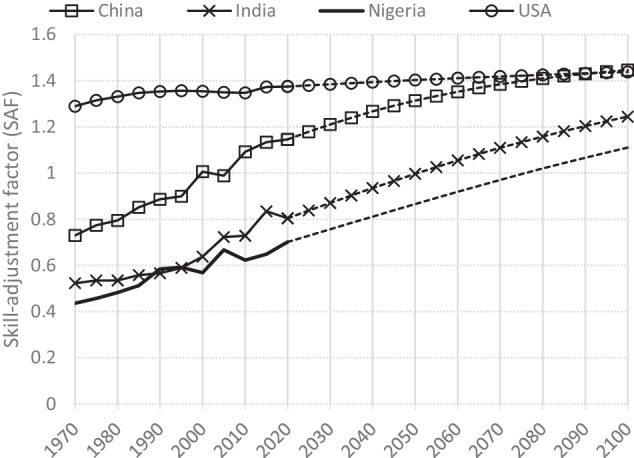
Skill-adjustment factor (1 = world average in 2015) for China, India, Nigeria and the US, estimated (1970–2015) and projected (2020–2100).

To adjust our education-weights for the quality of the education, we use these country- and period-specific SAF from 1970 to 2015 and perform a logit extrapolation for 2015–2100, with the maximum value being the world’s highest estimate in 2015 (which is 1.13 for Japan), thus leading to a gradual convergence towards the leading country. We then normalize the score to the world average of 2015. Figure 2 shows the estimated SAF for China, India, Nigeria and the US and the projections from 2020 to 2100.

SAF estimates are cross-sectional, without disaggregation by age, cohort, or specific level of education. In other words, using these values implies to assume no difference in the quality of education across the different degrees within a country. It is also assumed that the change in the cohort size of the population aged 20–64 has only a marginal effect on the trends. Finally, we use the simplifying assumption that the quality of education does not differ between those in the labor force and those out of the labor force.

### Population estimated and projected by age, sex, and education

The calculation of projected HCWP requires inputs from population projections by age, sex, and education. We used the most recent update of the population estimates (middle-of-the-road) for the period 1970–2015 and their population projection under five Shared Socioeconomic Pathways scenarios^25^. The last update of the projections and estimates take into account the impact of COVID-19 on the population structure as well as the latest estimates of demographic components as starting point. The Shared Socioeconomic Pathways scenarios for 2020–2100 are built around narratives and storyline describing alternative socio-economic developments. They were developed by climate scientists and economists to be used as a basis for integrated assessments and future climate modeling. The five scenarios are:

- SSP-1: Sustainability;
- SSP-2: Middle-of-the-road;
- SSP-3: Regional rivalry;
- SSP-4: Inequality;
- SSP-5: Fossil-Fueled Development.

The SSP-2 (Middle-of-the road) scenario can be considered as the reference scenario, as its assumptions are built based on historical patterns. For the mortality assumptions, 75 experts from 30 countries were asked for their opinion on a series of statements on past and future determinants of health and mortality. They concluded that there is room for improvement and that upward trends will continue, with convergence between countries. Country-specific trends are therefore extrapolated with a regional convergence process. Differentials by educational attainment are based on a generalization of estimates where such data are available. For the assumption on international migration, the scenario assumes a continuation of the in and out- migration rates of 1950–2010.

For education, final educational attainment (attained at age 30–34) is extrapolated by gender from country-specific estimates for 1970–2010 with a long-term world convergence. For those with a high level of education, lower levels are imputed at younger ages based on the country’s high school graduation age. In this projection, the educational attainment variable is categorical rather than in years of schooling. Therefore, education-weights are calculated based on the normal number of years of education for each level (see data source and sample selection). The future age- and education- specific fertility rates are determined on the basis of a large expert survey in the field of fertility studies^26,27^. The experts assumed a trend towards global convergence in the very long term. More details on the long-term assumptions and in the scenario definition can be found in in Lutz *et al*.^17,28^.

### Calculation of the human capital-weighted population

For a country *c* at time *t*, the human capital-weighted population (HCWP) is calculated as:

$$HCW{P}_{c,t}=SA{F}_{c,t}\mathop{\sum }\limits_{e=1}^{k}{W}_{e,c,t}POP206{4}_{e,c,t}$$

*W*_*e,c,t*_ corresponds to the education (e) weights specific to the average educational group of the country *c* at time *t* as calculated in the previous section. POP2064_e,c,t_ is the population aged 20–64 from country *c* at time *t* with an educational attainment *e*. SAF_c,t_ is the Skill-adjustment factor specific to country *c* at time *t*.

The population projection outcomes by education are sourced from the WIC (see previous section), in which the educational attainment variable is categorical rather than in number of years of schooling. Therefore, education-weights are calculated based on the assumed number of years of education for each level (see data source and selection of the sample). For instance, there were 76.4 M persons aged 20–64 with a postsecondary education (16 years of schooling) in USA in 2020 (POP2064_e6,USA,2020_). Since the average number of years of schooling in 2020 was 13.2, the value of W_e6,USA,2020_ is 2.45. Multiplying W_e6,USA,2020_ by POP2064_e6,USA,2020_, gives 187 M, which means, in other words, that these 76.4 M persons with upper secondary would have a human capital equivalent 187 M Americans who would have 9.3 years of schooling. The SAF value for USA in 2020 is 1.33. Adjusted for this factor, the human capital figure is 249 M. Assuming that within a country, the income is a good proxy for productivity, and that across countries, there is a linear relationship between the quality of education and productivity, these 76.4 M Americans with postsecondary education would have the productive capacity of 249 M persons with the 2015 world average’s human capital. The HCWP for USA in 2020 is given by the sum of this calculation for each educational group.

**Fig. 3:**
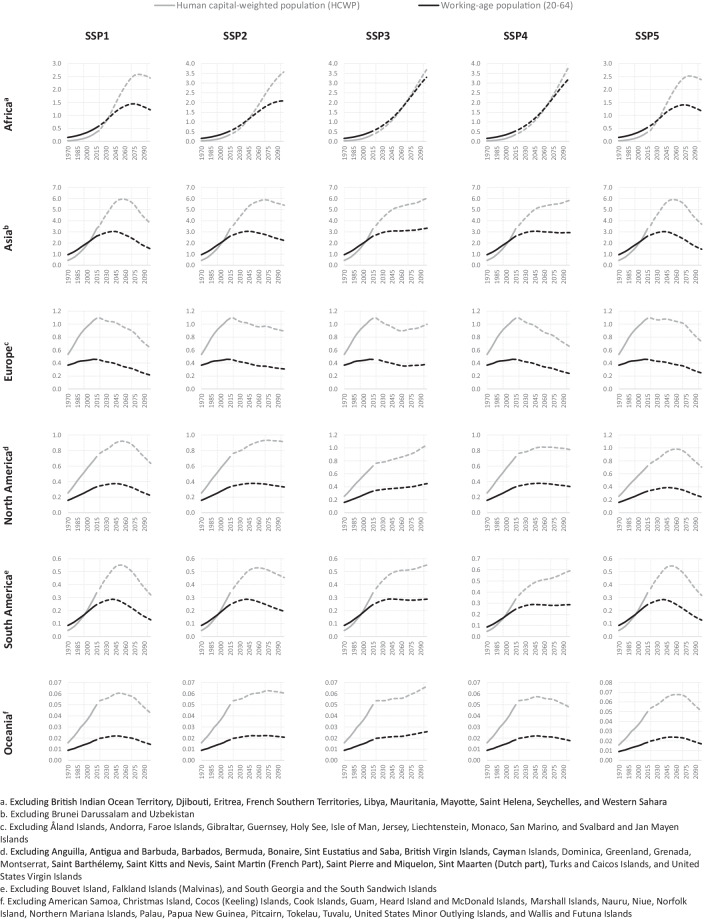
Working age population (WAP) and human capital-weighted population (HCWP), estimated (1970–2020) and projected (2020–2100) according to five SSP scenarios (in billions).

Figure 3 shows the estimated human capital-weighted population together with the working-age (20–64) population size from 1970–2020 by continent and their projection up to 2100 according to the five SSP scenarios.

## Data Records

The dataset OUTPUT_HCWP.csv available on Zenodo^1^ contains three columns for analytical indicators: human capital-weighted population (HCWP), total working-age population aged 20–64 (WAP) and the total population (POP) for 185 countries (identified in “CountryName” and “CountryCode” columns). The “CountryCode” column corresponds to country codes from the Standard Country or Area Codes for Statistical Use (M49) provided by the Statistics Division of the United Nations Secretariat. “Region” and “Continent” correspond to the geographical classification of countries by the United Nations Statistics Division.

The total working-age population and the total population are taken directly from the Wittgenstein Centre’s Data Explorer and are included in the dataset for information purposes, to give a better idea of the contribution of HCWP compared with this conventional indicator. Each variable contains 17,575 values, 185 countries X (10 periods from 1970–2015 + 5 scenarios X 17 periods from 2020 to 2100).

## Technical Validation

We validate the HCWP data in the context of out-of-sample economic growth forecasts, by comparing the predictive content of the information of HCWP with that of human capital variables based exclusively on educational attainment. We estimate the econometric model proposed by Crespo Cuaresma^5^ using data for GDP per capita and capital stocks from the Penn World Table 10.1^29^, population and educational attainment data from the WIC. The estimated models are based on an aggregate production function where technological progress is assumed to depend on innovation mechanisms (proxied alternatively using educational attainment and HCWP as the human capital stock variable) and technology adoption effects (included in the model as the interaction of the human capital variable and the level of income per capita). Using the estimated panel data models based on the global sample of countries and the period 1970–1995 in 5-year periods, out-of-sample predictions for all countries in 1995–2000 are obtained, and prediction error measures (root mean square error, mean absolute deviation and directional accuracy rate, that is, the share of correctly predicted directions of change) are computed. The exercise is repeated expanding the in-sample period to 1970–2000 and using the out-of-sample period 2000–2005, then for the in-sample period 1970–2005 and predictions for 2005–2010, and finally for models estimated using data for 1970–2010 and forecasts for 2010–2015. We compare the average prediction errors for the different models over all of the out-of-sample observations in this design.

The results of the validation exercise for the models including HCWP as a predictor, the specifications with educational attainment and a benchmark model where total population is used as a human capital variable (without heterogeneity based on education or productivity) are presented in Table 3. In the global sample, the results for the models based on HCWP are relatively similar to those based on educational attainment, but the specifications that use HCWP as an explanatory variable excel in prediction for the subsample of low and high-income economies. Models based on educational attainment, on the other hand, dominate in terms of forecasting ability for middle-income economies.

**Table 3:** Validation results based on predictive ability for GDP per capita growth.

	Educational attainment	HCWP	Benchmark
Global Sample	**0.235**/**0.174**/**0.744**	0.236/**0.174**/0.733	0.245/0.178/0.737
High Income	0.196/0.152/**0.653**	**0.186**/**0.143**/**0.653**	0.196/0.152/0.638
Lower Middle Income	**0.221**/**0.173**/**0.823**	0.226/0.179/0.814	0.224/0.176/**0.823**
Upper Middle Income	**0.278**/**0.198**/**0.772**	0.286/0.206/0.73	0.301/0.209/0.763
Low Income	0.257/0.183/0.748	**0.252**/**0.174**/**0.765**	0.267/0.186/0.757

Each cell presents the root mean square error (RMSE), mean absolute deviation (MAD) and directional accuracy rate (DA), with bold figures marking the best performer (RMSE/MAD/DA).

While the HCWP model offers a more comprehensive approach by additionally considering educational quality, the complex interplay between human capital accumulation and economic growth at the global level may limit the predictive ability of econometric models built upon the new data. In particular, the assumption of parameter homogeneity which underlies our econometric specification may be restrictive for our global sample of countries, that contains economies with very different institutional, political, and cultural characteristics. Furthermore, synergies between quantity and quality of education are not necessarily linear or additive, and exploring such interactions could be a promising path of future research. Finally, the data used to construct the HCWP model may contain inherent variability and measurement issues, particularly when assessing educational quality across different global contexts. Future research could delve deeper into context-specific analyses to further explore these dynamics.

## Usage Notes

The methodology used to produce the human capital-weighted population (HCWP) estimates has some limitations. First, human capital is assessed only in terms of the quality and quantity of education. This approach may oversimplify the multidimensional nature of human capital, as it fails to consider other critical dimensions such as health, which influences educational opportunities, labor force participation, and productivity^30^. By excluding health, our analysis could potentially overestimate the HCWP and underestimate cross-country variations. Indeed, the HCWP takes into account only the gains in human capital resulting from changes in education, neglecting the potential impact of health variables among others. Future research iterations should explore the inclusion of health-related factors to capture a more comprehensive understanding of human capital.

Moreover, the education variable itself may oversimplify the diversity in educational attainment, potentially masking important nuances in educational backgrounds. This approach may not fully capture the varying impacts of different types of education on human capital accumulation. This is particularly true in countries where a large share of the population has post-secondary education, which includes very different specializations and skills.

Furthermore, the use of pooled censuses from different countries and years to estimate human capital weights may overlook country-specific nuances and variations in educational systems, labor markets, and economic conditions, which could significantly influence the relationship between education and human capital. It is also worth noting that although the countries included in our sample cover many degrees of development, many countries are missing from the analysis. Most censuses were from countries in the Americas, potentially limiting the generalizability of our findings to other regions with distinct socioeconomic and cultural contexts.

Additionally, our approach of adjusting education weights for quality using cross-sectional data from adult literacy assessments assumes uniform quality across different levels of education within a country. This assumption may overlook potential variations in educational quality that could impact human capital differently across various educational levels.

Finally, projecting future human capital-weights based on assumptions about educational trends and fertility rates introduces uncertainties related to demographic changes, economic developments, and policy interventions that may not materialize as projected. These uncertainties could potentially affect the accuracy of our long-term projections.

## Data Availability

Regression models to estimate of human capital-weights (Eqs. 1, 2 and 3) were run using the GLIMMIX procedure in SAS 9.4. /*Model 1*/ proc glimmix data = data INITGLM; title "Fixed all"; class sample SEXR; model norm_sal = nbedu experience|experience SEXR / solution dist = gamma link = log; weight perwt; **where 0** < norm_sal < **15 and 20** < = agegr < = **64**; NLOPTIONS TECH = NRRIDG MAXITER = **100**; **run**; /*Model 2*/ proc glimmix data = data INITGLM; title "Random intercept"; class sample SEXR; model norm_sal = nbedu experience|experience SEXR / solution dist = gamma link = log; random intercept / subject = sample; weight perwt; **where 0 < norm_sal < 15 and 20 <** = agegr < = **64**; NLOPTIONS TECH = NRRIDG MAXITER = **100**; **run**; /*Model 3*/ proc glimmix data = data INITGLM; title "Random intercept with interaction"; class sample SEXR; model norm_sal = nbedu|nbedu_mean experience|experience SEXR / solution dist = gamma link = log; random intercept / subject = sample; weight perwt; **where 0 < norm_sal < 15 and 20 <** = agegr < = **64**; NLOPTIONS TECH = NRRIDG MAXITER = **100**; **run**; The code for the calculation of the Human capital weighted population estimates is available on Github (https://github.com/gmarois/HCWP/blob/main/Code%20for%20HCWP.sas).
